# Interleukin 1 blockade with canacinumab for hyperimmunoglobulin D and periodic fever syndrome

**DOI:** 10.1186/1546-0096-12-S1-P270

**Published:** 2014-09-17

**Authors:** Juergen Brunner, Elisabeth Binder, Daniela Karall, Johannes Zschocke, Christine Fauth

**Affiliations:** 1 Pediatrics, Medical University Innsbruck, Innsbruck, Austria; 2 Human Genetics, Medical University Innsbruck, Innsbruck, Austria

## Introduction

Hyperimmunoglobulinemia D and periodic fever syndrome (HIDS; MIM# 260920) is a rare autosomal recessive autoinflammatory condition caused by mutations in the *MVK* gene, which encodes for mevalonate kinase. There is no standard treatment for HIDS.

## Objectives

We report on a 2 year-old Austrian boy with recurrent episodes of fever, febrile seizures, arthralgias, and splenomegaly. Rash and abdominal pain were also seen occasionally. During attacks an acute-phase response was detected. Clinical and laboratory improvement was seen between attacks. These findings led to the tentative diagnosis of HIDS.

## Methods

Sequencing of the *MVK* gene showed a homozygous c.1129G>A (p.Val377Ile, also known as V377I) mutation in the child, while the healthy non-consanguineous parents were heterozygous. The mutation is known to be associated with HIDS.

## Results

Therapy with nonsteroidal anti-inflammatory drugs during attacks had poor benefit. A further febrile episode resulted in a status epilepticus. Treatment with canakinumab was initiated and a final dose of 4 mg/kg every 4 weeks resulted in the disappearance of febrile attacks and a considerable improvement of patient´s quality of life during a 6-month follow-up period. The drug has been well tolerated, and no side effects were observed.

## Conclusion

Treatment with canakinumab is a therapeutical option for patients with HIDS.

## Disclosure of interest

None declared.

